# Editorial: Crop-microorganisms interactions: diseases and symbioses

**DOI:** 10.3389/fmicb.2023.1225884

**Published:** 2023-06-30

**Authors:** Xiancan Zhu, Xiaojuan Tan, Fulai Liu, Jianfeng Wu, Liyan Song, Guoping Zhu

**Affiliations:** ^1^Anhui Provincial Key Laboratory of Molecular Enzymology and Mechanism of Major Diseases, College of Life Sciences, Anhui Normal University, Wuhu, China; ^2^Department of Plant and Environmental Sciences, Faculty of Science, University of Copenhagen, Copenhagen, Denmark; ^3^Department of Environmental Health Sciences, University of Michigan, Ann Arbor, MI, United States; ^4^School of Resources and Environmental Engineering, Anhui University, Hefei, China

**Keywords:** biological control, pathogen, arbuscular mycorrhizal fungi, abiotic and biotic stress, microbiome

Understanding the interactions between microorganisms and crops is essential for plant health and sustainable crop production. Both harmful and beneficial microorganisms have impacts on crops by influencing plant morphological features and their physiological and molecular processes. This Research Topic, “*Crop-microorganisms interactions: diseases and symbioses*,” comprises eight original research articles focusing on the molecular mechanisms of biocontrol against plant pathogens and the symbiotic functions of beneficial microbes in the agricultural ecosystem.

Plant pathogens pose a serious threat to crop growth and productivity. Tan et al. isolated and identified a pathogenic bacterium, the *Dickeya zeae* strain WH1, causing soft rot on rice roots. Comparative genomics demonstrated that WH1 has highly conserved plant cell wall-degrading extracellular enzymes and flagellar and chemotaxis and quorum-sensing systems. However, genes encoding zeamine biosynthesis, which is important for the virulence in *D. zeae* EC1 isolated from rice, were not found in the WH1 genome. Average nucleotide identity and DNA-DNA hybridization analyses showed that WH1 is phylogenetically closest to *D. zeae* EC2 isolated from rice. Pathogenicity assays revealed that WH1 induces severe soft rot symptoms in potato tubers, carrots, radishes, and Chinese cabbage. The results suggested that WH1 has likely developed an infection pathway different from the other *D. zeae* strains from rice, representing a new type of rice foot rot pathogen.

Some microbes and their secondary metabolites play a role against pathogenesis. Hu et al. isolated a *Bacillus velezensis* strain (YS-AT-DS1) from tidal soil and evaluated its biocontrol efficiency against the root-knot nematode (RKN) *Meloidogyne incognita* in tomato plants. They reported that this strain produces IAA and possesses antifungal and nematicidal activities, suggesting that *B. velezensis* has a dual effect in promoting plant growth and protecting the host against RKN. These results provide a theoretical reference for its application as a biocontrol agent for RKN control in sustainable agriculture. *Pseudomonas aeruginosa* notoriously affects food security and human health. In another work, Chen et al. highlighted the role of thymoquinone and nisin in the inhibition of biofilm formation by *P. aeruginosa* in lettuce plants. They found that the combination of thymoquinone and nisin reduced the virulence factors and inactivated the biofilm of *P. aeruginosa* on lettuce leaves. This study helps to better understand the anti-virulence and synergistic mechanisms of thymoquinone against *P. aeruginosa*.

Arbuscular mycorrhizal fungi (AMF) and endophytic fungi can establish symbiotic associations with plants, which play key roles in promoting plant growth and crop yield. Sun et al. studied the effects of AMF on the plant growth, phosphate (P) acquisition, medicinal component concentrations, and gene expressions of *Polygonum cuspidatum* at two P levels. Their results showed that AMF improved growth performance and root morphology, and the AM plants had higher root P concentration than the non-AM plants. The AMF inoculation and P supply increased the concentrations of chrysophanol, physcion, polydatin, and resveratrol, as well as the expressions of resveratrol synthesis-associated enzyme genes in *P. cuspidatum* plants. The conclusion revealed the mechanism by which AMF accelerates the production of medicinal components. In another paper, Li et al. investigated the effects of AMF on nitrous oxide (N_2_O) emissions and soil bacterial communities under varied precipitation conditions in a semiarid grassland. The results showed that the presence of AMF decreased N_2_O fluxes and altered soil bacterial community composition. The study highlighted that moderate soil moisture deficit promotes the function of AMF in reducing N_2_O emissions through regulating the N cycle in grassland. Furthermore, Rong et al. evaluated the effects of an endophytic fungus, *Serendipita indica*, on the metabolism of growth and reactive oxygen species in white clover under water stress. This fungus enhanced the plant biomass, chlorophyll content, and activities of superoxide dismutase, peroxidase, ascorbate peroxidase, and glutathione reductase. The results provide further understanding on the improvement of plant resistance to water stress by endophytic fungi via enhancing antioxidant enzyme activities and accelerating the ascorbate–glutathione cycle.

Agricultural management practices, such as mulching, tillage, and fertilization, influence soil microbial communities and quality, crop performance, and the functions of agroecosystems. In a long-term no-tillage study, Song et al. investigated the responses of the microbial functional guilds in the endosphere and rhizosphere as related to the frequency and amount of maize stover mulching. They found that the frequency and amount of maize stover mulching significantly affected the soil N cycle and the bacterial and fungal functional guilds in the roots and rhizosphere. They propose that stover mulch application twice every 3 years is the optimal mulching frequency to maintain plant health and N requirements in soils. In another long-term experiment, Wang et al. investigated the effects of straw returning and fertilizer application on soil fertility, root endophytic bacterial communities, and the occurrence of wheat crown rot. The results showed that fertilizer reduction coupled with straw returning decreased root endophytic bacterial diversity, changed the community structure and functions, and increased the relative abundance of potential biocontrol bacteria. They highlighted that this agricultural practice could maintain soil fertility and effectively reduce the occurrence of wheat crown rot. Both articles provide a theoretical basis for sustainable agricultural practices that reduce chemical fertilizers application and promote soil health and crop productivity.

In conclusion, plant pathogens cause diseases and adversely affect crop health and growth, while a number of beneficial microbes and other agents can control plant diseases and alleviate the adverse effects on plant physiology and growth. There is still a gap in the exploration of the direct or indirect interactions between pathogens and beneficial microbes on crops. We hope this article collection prompts frontline researchers to go further into this research area, contributing to the improvement of the resilience of crops to abiotic and biotic stresses, soil health, food security and sustainable agricultural development.

## Author contributions

All authors listed have made a substantial, direct, and intellectual contribution to the work and approved it for publication.

